# Optimization of growth of *Levilactobacillus brevis* SP 48 and *in vitro* evaluation of the effect of viable cells and high molecular weight potential postbiotics on *Helicobacter pylori*

**DOI:** 10.3389/fbioe.2022.1007004

**Published:** 2022-10-31

**Authors:** Donatella Cimini, Sergio D’ambrosio, Antonietta Stellavato, Alessandra Fusco, Maria Michela Corsaro, Azza Dabous, Angela Casillo, Giovanna Donnarumma, Andrea Maria Giori, Chiara Schiraldi

**Affiliations:** ^1^ Department of Environmental, Biological and Pharmaceutical Sciences and Technologies, University of Campania Luigi Vanvitelli, Caserta, Italy; ^2^ Department of Experimental Medicine, University of Campania “L.Vanvitelli”, Naples, Italy; ^3^ Department of Chemical Sciences, University of Naples “Federico II”, Complesso Universitario Monte S. Angelo, Naples, Italy; ^4^ R&D—IBSA Farmaceutici Italia, Lodi, Italy

**Keywords:** *Levilactobacillus brevis*, probiotic, postbiotic, exopolysaccharide, *Helicobacter pylori*, gut model

## Abstract

Several *Levilactobacillus brevis* strains have the potential to be used as probiotics since they provide health benefits due to the interaction of live cells, and of their secreted products, with the host (tissues). Therefore, the development of simple fermentation processes that improve cell viability to reduce industrial production costs, and at the same time the characterization and biological evaluation of cell-free postbiotics that can further promote application, are of great interest. In the present study, small scale batch fermentations on semi defined media, deprived of animal derived raw materials, were used to optimize growth of *L. brevis* SP48, reaching 1.2 ± 0.4 × 10^10^ CFU/ml of viable cells after 16 h of growth. Displacement, competition, and inhibition assays compared the effect, on *Helicobacter pylori*, of *L. brevis* cells to that of its partially purified potentially postbiotic fraction rich in exopolysaccharides and proteins. The expression of pro and anti-inflammatory biochemical markers indicated that both samples activated antimicrobial defenses and innate immunity in a gastric model. Moreover, these compounds also acted as modulators of the inflammatory response in a gut *in vitro* model. These data demonstrate that the high molecular weight compounds secreted by *L. brevis* SP48 can contrast *H. pylori* and reduce inflammation related to intestinal bowel disease, potentially overcoming issues related to the preservation of probiotic viability.

## 1 Introduction

Besides targeting gastrointestinal disorders probiotics such as some lactobacilli have been reported to improve immunomodulation and protection of the host against infections caused by viral and bacterial pathogens (Di Cerbo et al., 2016). Interestingly, also inactivated microbial (probiotic) cells and their metabolic products, indicated as parabiotics and postbiotics (Aguilar-Toala et al., 2018), respectively, are crucial and act as mediators in the modulation of the host’s immune function (Teame et al., 2020). Moreover, the use of cell structural components and metabolites reduces safety concerns regarding potential theoretical risks that have been described in specific conditions in relation to live probiotic administrations (e.g. experimental models, systemic infections, deleterious metabolic activities, excessive immune stimulation in susceptible individuals, gene transfer and gastrointestinal side effects) (Doron and Snydman, 2015). Therefore overall, health preservation can be improved on the one side by the direct competition of probiotics with pathogens through nutrient subtraction and production of antimicrobial and immunostimulatory molecules (postbiotics), and on the other hand by the interaction of specific parabiotics with human cells *via* mucosa-associated lymphoid tissue, resulting in the activation of the immune system (Taverniti and Guglielmetti, 2011).

*Levilactobacillus brevis* (previously identified as *Lactobacillus brevis*) is an obligate heterofermentative LAB isolated from plants, food, and human microbiota. In addition to common and demonstrated probiotic properties (Kishi et al., 1996; Suzuki et al., 2013; Abdelazez et al., 2018) several *L. brevis* strains also possess other features. *L. brevis* NCL912 and *L. brevis* CGMCC1306, for instance, have the natural ability to produce *γ*-aminobutyric acid (GABA), a neurotransmitter that reduces the threat of type 1 diabetes (Wu and Shah, 2017; Lyu et al., 2018; Wang et al., 2018; Wu and Shah, 2018). *L. brevis* SP48 is present in commercial products (e.g. Florap lady) designed for women to promote the balance of the intestinal flora and the functionality of the urinary tract. It was also evaluated as a tool to target the dysbiosis associated with periodontal diseases (Giannini et al., 2022).

Among the array of molecules produced by LAB, polysaccharides attached on the cell surface or secreted in the medium from numerous probiotic strains showed a wide range of properties as antitumor, immunostimulatory, cholesterol-lowering, and promoting adhesion to intestinal epithelial cells (Živković et al., 2016; Wei et al., 2019; Butorac et al., 2021). Those produced by *L. brevis* KB290 and *L. brevis* CD2 demonstrated to have a critical role in increasing cell-mediated cytotoxic activity in mouse spleen (Sasaki et al., 2015) and enhancing inhibition of the gut pathogen *Salmonella typhimurium,* respectively (Alfano et al., 2020). The composition of the exopolysaccharides (EPS) produced by few *L. brevis* strains has been previously described suggesting the polysaccharide to be either a glucose homotype or a glucose and N-acetylglucosamine heterotype polymer (Suzuki et al., 2013; Fraunhofer et al., 2018; Fukao et al., 2019).

To enhance the production of viable biomass and/or the synthesis of potentially bioactive compounds, such as EPS, efficient fermentation processes are necessary. Batch and fed-batch experiments on semidefined media previously investigated the final concentration of biomass, in terms of viable cells, and the production of related products focusing on the pH, the aeration, and on the feeding strategy (Martesson et al., 2003; Brooijmans et al., 2009; Alfano et al., 2020). Also, the simplification of the cultivation medium and the reduction of cultivation time are crucial aspects, especially considering potential industrial development. Another issue regards the identification of simple and industrially suitable purification processes to obtain bioactive postbiotic fractions applicable in the biomedical field.

In this regard the aim of the present study was to investigate growth of *L. brevis* SP48 in 2l-batch processes to favor the production of biomass and live cells and to assess whether a simple and industrially applicable downstream strategy could provide potentially bioactive secreted fractions.

Recent literature reports that extracellular polysaccharides can modulate intestinal immune responses by regulating Toll-like receptors (e.g TLR-2 and 4) signaling pathways, which are responsible for the induction of cytokines and chemokines in response (Wachi et al., 2014).

We here investigated the anti-inflammatory effects of secreted potential postbiotic fractions, mainly composed of EPS and secreted proteins, recovered from the *L. brevis* fermentation broth, on human enterocytes obtained by the differentiation of Caco-2 cells. The potentially bioactive compounds were also evaluated on a gut inflammation model (using LPS from *Salmonella minnesota*) exploiting both enterocytes and NF-κB reporter THP-1 cells.

*L. brevis* CD2 was previously shown to reduce the intragastric load of *Helicobacter pylori* (Linsalata et al., 2004), and to inhibit oral pathogens causing mucositis and periodontal inflammation (Sharma et al., 2017). Therefore, in the present work a comparison of the antimicrobial effect of live cells and of the secreted potential postbiotics on *H. pylori* (ATCC) was investigated as well as the composition of the polysaccharides present in the partially purified supernatant by nuclear magnetic resonance (NMR), gas chromatography-mass spectrometry (GC-MS) and high-performance anion exchange chromatography (HPAE-PAD).

## 2 Materials and methods

### 2.1 Bacterial strains and media

*L. brevis* SP48 (DSM deposit number 16806) was provided lyophilized by the “Centro Sperimentale del latte S.r.l.-” and stored at 4°C. Stocks were prepared from exponentially growing cells in Man, Rogosa and Sharpe (MRS) broth and conserved at −80°C in 20% v/v glycerol.

Human gastric adenocarcinoma cell-line AGS cells ATCC® CRL-1739™ (American Type Culture Collection, Rockville, MD, United States), were routinely cultured in Ham’s F-12K medium (Gibco, Waltham, Massachusetts,United States) supplemented with 1% Penstrep, 1% glutamine and 10% fetal calf serum (Gibco) at 37°C at 5% CO_2_.

*Helicobacter pylori* ATCC® 43629™ was cultured on Trypticase Soy Agar (Oxoid; Unipath, Basingstoke, United Kingdom) at 37°C under microaerophilic conditions for 2–4 days (as described on the ATCC website). *L. brevis* before use was cultured in MRS anaerobically at 37°C for 24–48 h, centrifuged at 4,000 × g, for 10 min, at 4°C, washed twice with saline, resuspended at the concentration of 0.5 OD in DMEM without antibiotics and added to cell cultures.

All medium components and salts were supplied by Sigma-Aldrich (St. Louis, MO, United States). Yeast extract was furnished by Organotechnie (La Corneuve, France), while sulphuric acid was purchased by Biochem s.r.l. (Turin, Italy). The semi-defined media used for growth experiments contained per liter: yeast extract 10 g, soy peptone 10 g, MgSO₄* 7 H₂O, 0.25 g, MnSO₄*H₂O 0.05 g, Na_3_C_6_H_5_O_7_ 2 g, Tween 80 0.45 ml, L-ascorbic acid 0.5 g and NaCl 0.2 g. Glucose and fructose (Sigma-Aldrich, St. Louis, MO, United States) used as carbon sources, were filter sterilized and added to all semi-defined media after autoclaving.

### 2.2 Bioreactor experiments

Fermentation experiments were performed in a Biostat CT plus (Sartorius Stedim, Gottingen, Germany) bioreactor with a working volume of about 2.2 l. Temperature was controlled at 32°C, pH at 6.1 and agitation was fixed at 150 rpm. Batch experiments were conducted on a semidefined medium described in the previous paragraph. Fructose (30 ± 3 g/l) and glucose (32 ± 4 and 43 ± 2 g/l) were used as carbon sources. For batch experiments with 58 ± 1 g/l of glucose, the concentration of complex nitrogen sources was increased to 15 g/l of soya peptone and 20 g/l of yeast extract in the medium. Before each experiment, a concentrated stock solution (20 OD_600_, 2% v/v) was inoculated in 0.45 l medium and incubated at 32°C and 150 rpm for 8 h. A peristaltic pump (model 313 U, Watson-Marlow, England) was used to transfer the pre-culture inside the vessel reaching up to 10% (v/v) of the working volume inside the fermenter. The airflow was kept constant at 0.44 vvm, and stirring was set to 150 rpm. Experiments lasted from 16 to 19 h. Each fermentation experiment was performed at least in triplicate.

Dry cell weight was evaluated by centrifuging 50 ml of broth in a falcon tube at 5,000 × g at 4°C for 30 min. After discarding the supernatant, the pellet was washed with 0.5 volumes of saline solution and then transferred in a pre-weighted Eppendorf tube. The pellet was dried o/n at 70°C before measuring the dry weight. Viability was evaluated by serially diluting the samples and plating on MRS-agar medium. Plates were incubated at 37°C for 36 h before counting viable cells. Each sample was analyzed in triplicate.

### 2.3 HPLC quantification of sugars, organic acids, and ethanol

Broth samples of about 10 ml were withdrawn during the experiments (at the time zero and after 16, 17, 18, and 19 h for fermentations on media 1 and 2, and only at time zero and after 16 h for fermentations on medium 3) for HPLC analyses of substrate consumption and acid production. The broth was centrifuged for 15 min at 10,000 ×g to separate the biomass and recover the supernatant. One ml was then UF/DF on 3 kDa centrifugal filter devices (Centricon, Amicon, and Sigma-Aldrich) at 10,000 ×g and the permeate was analysed for the determination of the concentrations of fructose, glucose, lactic and acetic acids and ethanol by HPLC (UHPLC Dionex Ultimate 3,000; Thermofisher) on a Alltech IOA-2000 column (500 mm × 6.5 mm ID) as previously reported (D’ambrosio et al., 2021).

### 2.4 Purification of potentially bioactive secreted fractions and extraction of the cell-bound polysaccharide

The broth collected at the end of the batch processes was centrifuged at 5,000 ×g for 30 min at 4°C to separate biomass and supernatant. The supernatant was ultrafiltrated on 10 kDa cut-off polyether sulfone membranes (GE Healthcare, Illinois, United States) with a filtering area of 0.1 m^2^. Tangential flow filtration was performed on a Sartoflow alpha (Sartorius Stedim, Gottingen, and Germany) system connected with a thermostatic bath that kept a temperature of about 20–25°C. The retentate was concentrated about 6 fold and washed with 3 volumes of milliQ water to remove low molecular weight molecules still attached to the membrane, and salts. The retentate was then precipitated with 2 volumes of a 1:1 ethanol:acetone solution, after adjusting the conductivity to 20 mS/cm, and dried in a vacuum oven at 40°C over-night. The obtained powder was then suspended at 30 g/l in milliQ water and treated with 1% (w/v) of activated charcoal (Supelco Analytical, Sigma-Aldrich, Missouri, United States) in batch for 1 h at room temperature. The suspension was filtered on Velapad 60 filtration system (PALL Corporation, Milan, Italy) with nitrocellulose filters (BECO—PR Steril S80) purchased from Fluxa Filtri (Milan, Italy). The solution was precipitated, and the pellet was dried as described above. The recovered powder was used for assays described in the following paragraphs. Protein concentration was determined by Bradford assays (Bradford 1976).

By slightly modifying the protocol reported by Tallon and collaborators (2003) the cell-bound portion of the exopolysaccharide was extracted from *L. brevis* SP48 biomass obtained from fermentation processes. Briefly, 25 ml of broth were centrifuged at 6,500 rpm for 10 min at 4°C, the pellet was washed twice with sterile saline, and suspended it in 5 ml of EDTA 5 mg/ml (Sigma-Aldrich, MO, United States) and stirred gently for 4 h at 4°C. After incubation the suspension was centrifuged as described above, and the pellet was suspended in 10 ml of Dulbecco’s Phosphate-Buffered Saline (PBS, Gibco, California, United States). Two volumes of chilled ethanol (Sigma-Aldrich, MO, United States) were added and incubated at 4°C overnight. The suspension was centrifuged (6,500 rpm, 10 min, 4°C) and the precipitate was suspended in 10 ml of distilled water and dialyzed against 3 l of distilled water using a 3.5 kDa dialysis membrane (Delchimica Scientific Glassware, Napoli, Italy) for 2 days with three water changes per day. The recovered solution was lyophilized in a Beta 2-8 LD Plus Freeze Dryer (Christ, Osterode am Harz, Germany). The powder was suspended in 50 mmol/l Tris–HCl buffer at pH 8.6 (Sigma-Aldrich, MO, United States) and treated with 2 μg/ml DNase (Sigma-Aldrich, MO, United States) and 0.2 μg/ml RNase (Sigma-Aldrich, MO, United States) at 37°C for 6 h and following it was treated with 20 μg/ml proteinase K (Sigma-Aldrich, MO, United States) at 37°C for 16 h. The reaction was stopped by heating the solution at 95°C for 10 min. Ethanol precipitation and dialysis were repeated as described above. The obtained sample was dried in a vacuum oven (OV-11, Thermo Fisher Scientific, MA, United States) at 40°C overnight. This sample was hydrolysed and analysed by HPAE-PAD as described in paragraph 2.6.

### 2.5 Phenolsulfuric acid assay for exopolysasccharides quantification

Quantification of polysaccharides was performed by phenolsulfuric acid test (Dubois et al., 1956), a colorimetric assay used for the determination of total sugars in a sample. During hydrolysis pentoses are dehydrated to furfural, and hexoses to hydroxymethylfurfural producing a yellow-gold color in presence of phenol. The calibration curve was obtained with standard solutions of D (+) glucose at concentrations ranging from 0.01 to 0.1 mg/ml. Briefly, 200 μl of standards/sample were placed in a reaction tube with 200 μl of aqueous solution of phenol 5% w/v. Then, 1 ml of concentrated sulfuric acid (98% w/w) was added and the reaction tube was quickly closed and after vigorous stirring, the reaction was carried out for 30 min at 30°C. Sample absorbance was read at 490 nm. The blank was obtained by adding water to the reaction mixture.

### 2.6 EPS characterization by NMR, GC-MS, and HPAEC-PAD

Mono- and two-dimensional NMR spectra were recorded at 298 K, dissolved in D_2_O (sample concentration 2 mg/ml), by using a Bruker Avance-600 (^1^H: 600 MHz, and ^13^C: 150 MHz) spectrometer as reported previously (Fresno et al., 2007).

To determine the monosaccharide composition 1 mg of the sample deriving from the partial purification of the fermentation supernatant (containing potential postbiotics) was subjected to a methanolysis reaction with 1.25 M MeOH/HCl (80°C, 16 h), to obtain the *O*-methyl glycosides (Casillo et al., 2021). The methanol layer was extracted three times with hexane to remove the fatty acids eventually present, and dried. Then the *O*-methyl glycosides were acetylated with 25 μl of acetic anhydride and 25 μl of pyridine (100°C for 30 min). The acetylated methyl glycosides were dissolved in acetone and analysed by using an Agilent Technologies gas chromatograph 7820A equipped with a mass selective detector 5977B and a HP-5ms capillary column (Agilent, Italy 30 m × 0.25 mm i. d., flow rate 1 ml/min, He as carrier gas). The analysis was performed by using the following temperature program: 140°C for 3 min, then 140 → 240°C at 3 C/min.

The linkage pattern of the EPS was obtained by analyzing the partially methylated and acetylated alditol derivatives of the monosaccharide units. The linkages of the monosaccharides were recognized by their mass spectra fragmentation and by comparison of their retention times with those of authentic standards. The methylation reaction was carried out by using Ciucanu and Kerek procedure (Ciucanu and Kerek, 1984). The methylated EPS was hydrolyzed with trifluoroacetic acid, reduced with NaBD_4_, and acetylated with 25 μl of acetic anhydride and 25 μl of pyridine (100°C for 30 min). The partially methylated acetylated alditols dissolved in acetone were analyzed by GC–MS using the following temperature program: 90°C for 1 min, 90°C → 140°C at 25 C/min, 140°C → 200°C at 5 C/min, 200 °C → 280°C at 10 C/min, 280°C for 10 min. HPAEC-PAD (model ICS- 3,000, Dionex, CA, United States) was used to analyse the monosaccharides obtained after hydrolysis of the other partially purified fermentation supernatant containing the high molecular weight potentially bioactive compounds, on the treated unspent medium, and on the cell-bound polysaccharide sample obtained as described in paragraph 2.4. Before the analyses, samples (20 mg/ml powder) were hydrolysed in HCl 5 M for 6 h at 100°C. Diluted samples were analysed on a CarboPac PA10 column (4 × 250 mm) equipped with a guard column (4 × 50 mm) (Dionex, CA, United States) at a flow rate of 1 ml/min. The solutions used for the gradient are the following: 1) 1 mM NaOH and 1 M NaAcOH, 2) 1 mM NaOH. Analytes were detected by a pulsed amperometric detector (reference electrode Ag–AgCl; working electrode Au) and quantified using external standard calibration curves. Standard concentrations were linear from 0.008 to 0.0005 mg/ml for Glucose, Galactose and Mannose.

### 2.7 Infection of AGS cells with *H. pylori* and/or *L. brevis* and/or secreted potential postbiotics and evaluation of proinflammatory gene expressions

The ability of *L. brevis* SP48 to reduce the inflammation in AGS (human gastric adenocarcinoma) cells after infection with *H. pylori*, was investigated in three different experimental set ups: 1) competitive assay, in which AGS cells (10^5^) were incubated simultaneously with *L. brevis* (10^8^ CFU/ml) and *H. pylori* (10^8^ CFU/ml) for 2 h; 2) inhibition assay, in which AGS cells (10^5^ cells) were preincubated with (10^8^ CFU/ml) for 1.5 h and then *H. pylori* (10^8^ CFU/ml) was added and incubated for 2 h; 3) displacement assay in which AGS were pre-incubated with *H. pylori* (10^8^ CFU/ml) for 2 h and then *L. brevis* (10^8^ CFU/ml) was added and further incubated for 1.5 h (Kui et al., 2018). Semi confluent monolayers of AGS cells, cultured as previously described, were infected with *H. pylori* (10^8^ CFU/ml) in the presence of postbiotics produced from *L. brevis,* purified as described in paragraph 2.4, at 200 μg/ml and 600 μg/ml of powder dissolved in PBS for 4 h. At the end of the experiments, in order to evaluate the expression of pro- and anti-inflammatory cytokines, the cells were washed three times with sterile PBS, and the total RNA was extracted using High Pure RNA Isolation Kit (Roche Diagnostics, Monza, and Italy). Two hundred nanograms of total cellular RNA were reverse-transcribed (Expand Reverse Transcriptase, Roche Diagnostics) into complementary DNA (cDNA) using random hexamer primers (Random hexamers, Roche Diagnostics) at 42°C for 45 min, according to the manufacturer’s instructions [19]. Real time PCR for IL-6, IL-8, TNF-α, IL-1α, TGF-β, and HBD-2 was carried out with the LC Fast Start DNA Master SYBR Green kit using 2 µl of cDNA, corresponding to 10 ng of total RNA in a 20 ml final volume, 3 mM MgCl_2_ and 0.5 mM sense and antisense primers (Table 1). After amplification, melting curve analysis was performed by heating to 95°C for 15 s with a temperature transition rate of 20°C/s, cooling to 60°C for 15 s with a temperature transition rate of 20°C/s, and then heating the sample at 0.1°C/s to 95°C. The results were then analyzed using LightCycler software (Roche Diagnostics). The standard curve of each primer pair was established with serial dilutions of cDNA. The relative gene expression was measured by adding a sample at known concentration of the standard curve in each test carried out. All PCR reactions were run in triplicate. The specificity of the amplification products was verified by electrophoresis on a 2% (w/v) agarose gel and visualization by ethidium bromide staining.

**TABLE 1:** Primer sequences and amplification programs used in the studies on AGS and Caco-2 cell cultures.

Gene name	Primer sequence 5′→ 3′	Conditions	Product size (bp)
**Expression of proinflammatory genes in AGS cells**			
IL-6	ATG​AAC​TCC​TTC​TCC​ACA​AGC​GC	5″at 95°C, 13″ at 56°C, 25″at 72°C for 40 cycles	628
	GAA​GAG​CCC​TCA​GGC​TGG​ACT​G		
IL-8	ATG​ACT​TCC​AAG​CTG​GCC​GTG	5″at 94°C, 6″ at 55°C, 12″at 72°C for 40 cycles	297
	TGA​ATT​CTC​AGC​CCT​CTT​CAA​AAA​CTT​CTC		
TGF-β	CCG​ACT​ACT​ACG​CCA​AGG​AGG​TCA​C	5″at 94°C, 9″ at 60°C, 18″at 72°C for 40 cycles	439
	AGG​CCG​GTT​CAT​GCC​ATG​AAT​GGT​G		
IL-1α	CAT​GTC​AAA​TTT​CAC​TGC​TTC​ATC​C	5″at 95 °C, 8″at 55 °C, 17″at 72 °C for 45 cycles	421
	GTC​TCT​GAA​TCA​GAA​ATC​CTT​CTA​TC		
HBD-2	GGA​TCC​ATG​GGT​ATA​GGC​GAT​CCT​GTT​A	5″at 94°C, 6″at 63°C, 10″at 72°C for 50 cycles	198
	AAG​CTT​CTC​TGA​TGA​GGG​AGC​CCT​TTC​T		
TNF-α	CAG​AGG​GAA​GAG​TTC​CCC​AG	5″at 95°C, 6″ at 57°C, 13″at 72°C for 40 cycles	324
	CCT​TGG​TCT​GGT​AGG​AGA​CG		
**Expression of inflammatory biomarkers in differentiated Caco-2**			
GAPDH	TGC​ACC​ACC​AAC​TGC​TTA​GC GGC​ATG​GAC​TGT​GGT​CAT​GAG	3′ at 95°C, 10″at 95°,30″ at 55°C, 30″at 55°C, 10″ at 55°C for 40 cycles	280
TLR-4	TCC​CAG​GAA​TTG​GTG​ATA​AAG​TAG​A		
	CTG​GCA​TGA​CGC​GAA​CAA​TA	3′ at 95°C, 10″at 95°,30″ at 55°C, 30″at 55°C, 10″ at 55°C for 40 cycles	255
IL-6	GTG​GAG​ATT​GTT​GCC​ATC​AAC​G CAG​TGG​ATG​CAG​GGA​TGA​TGT​TCT​G	3′ at 95°C, 10″at 95°,30″ at 55°C, 30″at 55°C, 10″ at 55°C for 40 cycles	301

### 2.8 ELISA assay

Supernatants of AGS cells infected as described above at the end of the experiment, were harvested and the presence of cytokines IL-6, IL-8, IL-1α, TNF-α, TGF-β, and HBD-2 was analyzed by enzyme-linked immunosorbent assay (ELISA; ThermoFischer Scientific Inc., Waltham, Massachusetts,United States; Phoenix Pharmaceuticals, Burlingame, United States).

### 2.9 Differentiated Caco-2 cell culture and treatment conditions, and TLR-4 and IL-6 mRNA analyses by qRT-PCR

The *in vitro* model presented was used in a previous study from our group, (Di giuda et al., 2021) and here slightly modified. Specifically, Caco-2 cells (Human Caucasian colon adenocarcinoma cells, ATCC® HTB-37™) were cultivated in Dulbecco’s modified eagle medium (DMEM) containing glucose and glutamine and supplemented with 10% (v/v) fetal bovine serum (FBS) heat inactivated (56°C for 30 min). The cells were grown in a sterile 25 cm^2^ flask at a concentration of 3 × 10^5^ at confluence for 21 days to reach differentiation in normal enterocytes (Fusco et al., 2021). THP1-Blue™ NF-κB reporter cells were purchased by *In Vivo* Gen (Toulouse, France) and maintained in Roswell Park Memorial Institute (RPMI) 1,640 medium containing glucose (2 g/l) and glutamine (0.3 g/l), supplemented with 10% (v/v) FBS, 100 μg/ml, Normocin™, and Pen-Strep (100 U/ml). Cells were incubated at 37°C in a humidified atmosphere of air/CO_2_ (95:5, v/v). Total RNA was isolated by cells using TRIzol RNA Isolation Reagents (Thermo Fisher Scientific, Waltham, MA, United States) and subsequently the cDNA was reverse transcribed by Reverse Transcription System Kit (Promega, Milan, and Italy) according to the manufacturer’s instructions. Quantitative real-time polymerase chain reactions (qRT-PCR) were performed in duplicate for all genes of interest using IQ ™ SYBR® Green Supermix (Bio-Rad Laboratories, Milan, Italy) and as internal control the glyceraldehyde-3-phosphate dehydrogenase (GAPDH) housekeeping gene. The primers of the target genes are reported in Table 1. The data are expressed as a fold change (2^−∆∆Ct^ method), (Livak and Schmittgen, 2001) in treated cells vs. untreated cells (the control) and normalized to transcript levels of housekeeping gene (Stellavato et al., 2020). To analyze the anti-inflammatory properties of *L. brevis* SP48 alone and of the secreted high molecular weight fraction (potential postbiotics), human enterocytes treated with LPS were used as cellular model of inflammation. Specifically, different experimental set ups were exploited in order to compare: 1) LPS treated (20 μg/ml) and untreated cells (CTR), 2) *L. brevis* alone (1 × 10^6^ CFU/ml), 3) the combination LPS *+ L. brevis*, iiii) postbiotics produced by *L. brevis* alone, and LPS + postbiotics at the final concentration of 200 μg/ml.

### 2.10 Western blotting for TLR-4, NF-κB, and tubulin

Western blotting analyses were performed after 48 h of treatment. Ripa buffer (Cell Signaling Technology) was used to lyse cells. The intracellular protein concentration was quantified using the Bradford method. In particular, 30 µg of proteins for each sample were resolved on a 10% SDS–PAGE gel and transferred onto nitrocellulose membrane (GE, Amersham, United Kingdom). Following, the membrane was blocked by 5% w/v non-fat milk in Tris-buffered saline and 0.05% v/v Tween-20 (TTBS) for 30 min and incubated overnight at 4°C with primary antibodies against TLR-4 (Abcam, Cambridge, United Kingdom) and NF-κB (Santa Cruz, Dallas, TX, United States) diluted 1:500. The membrane was then incubated with secondary antibodies, horseradish peroxidase-conjugated donkey anti-rabbit and goat anti-mouse, which was diluted 1:5,000 for 2 h at room temperature. Anti-β-tubulin antibody diluted 1:1,000 was used as the loading control. The signal was detected using the ECL system (Chemicon-Millipore, Milano, Italy), and the semi-quantitative analyses of protein expression were carried out with the ImageJ program.

### 2.11 QUANTI-THP-1 blue assay using Caco-2/THP-1 co-culture

Caco-2 monolayers were cultured to confluence (triplicates for each condition) for 21 days to reach full differentiation according to previously reported protocols (Fusco et al., 2021). In order to obtain a gut inflammation model, these monolayers were incubated with *L. brevis* EPS alone (200 μg/ml), and in the presence of commercial LPS (LPS from *Salmonella minnesota* S-form, Enzo Life Sciences, Farmingdale, NY, United States) at 20 μg/ml for 24 h. Successively, THP1-Blue™ (5 × 10^4^ cells/ml) were added to all the treated monolayers according to a recently reported co-culture model (Calatayud et al., 2021). Supernatants were collected after 6 h incubation for NF-κB quantification (QUANTI-Blue reagent, InvivoGen, Toulouse, France). The assay was performed following the manufacturer’s instructions, and luminescence intensity was quantified at 655 nm using a Microplate Reader (Biorad Laboratories, Milan, Italy) to determine SEAP (secreted embryonic alkaline phosphatase) levels.

### 2.12 Statistical analyses

Data generated during the fermentation processes were analyzed by means one way ANOVA (JASP) with post-hoc comparisons (Tukey, Boferroni, Scheffe, Holm, and Sidak). *p* < 0.05 was considered as statistically significant. For cultivations of AGS cells with *H. pylori* and *L. brevis*, significant differences among groups were assessed by two-way ANOVA using GraphPad Prism 6.0, and the comparison between the means was calculated by t-student test. Data are expressed as means ± standard deviation (SD) of three independent experiments.

## 3 Results

### 3.1 Production of biomass by fermentation

Batch processes were performed initially on a medium containing high concentrations of glucose (58 ± 2 g/l) and complex nitrogen (yeast extract and soy peptone) to support the production of viable *L. brevis* cells and postbiotics. In this condition, glucose was almost completely consumed and 6.3 ± 0.2 g_cdw_/l of dry biomass and 9.1 ± 0.4 × 10^9^ CFU/ml were obtained, accompanied by about 41.0 ± 5.0 g/l of lactic acid (Table 2). A reduction of the glucose concentration, by 25 and 40%, respectively, combined with only half of the complex nitrogen sources lowered significantly the titer of dry biomass (*p* < 0.001) and of LA (*p* < 0.01), although it did not decrease the concentration of viable cells (Table 2) and of EPS, that was not significantly different in the three media. Growth of *L. brevis* on medium 3 with fructose as main carbon source reduced the production of viable cells and the concentration of dry biomass (*p* < 0.001) that was in fact 2.2 and 1.7-fold higher on the same medium containing glucose as carbon source (Table 2). Growth on fructose was accompanied by a high residue of unconsumed carbon (14.0 ± 0.2 g/l) which also led to a significantly higher Y_x/s_ (Table 2, *p* < 0.001). Overall ANOVA data analysis highlighted a significative increase of the production of viable cells in the presence of glucose, whereas similar results were obtained by changing the concentration of glucose itself and of complex nitrogen sources, with an average production of 1.02 ± 0.35 × 10^10^ CFU/ml. Interestingly fructose produced very similar C moles of lactic and acetic acid, whereas in the presence of glucose the spectrum of by-products changed in favor of lactic acid and ethanol, increasing the Y_LA/X_ yield up to 3.5 fold. Moreover, EPS values were below the sensitivity threshold on the medium containing fructose.

**TABLE 2:** Batch experiments performed on a Biostat CT plus reactor with a working volume of 2.2 l at 32°C, on semidefined medium with different carbon sources and amounts of complex nitrogen sources. n.d. not detected, below assay threshold. Mean and standard deviation were calculated from three biological replicates for each condition.

Fermentation medium	Viability (CFU/ml)	Biomass (g_cdw_/l)	Y_x/s_ (g/g)	Y_LA/x_ (g/g)	Y_LA/s_ (g/g)	LA (g/l)	AA (g/l)	Et (g/l)	LA:AA:Et (Cmoles)	EPS (Glucose equivalents) (g/l)
1) Glucose 60 g/l, 20 g/l YE, 15 g/l soya	9.1 ± 0.4 ×10^9^	6.3 ± 0.2^$3,*3,°3^	0.11 ± 0.00^°1^	6.54 ± 0.61°^2^	0.74 ± 0.09°^2,$1^	41.0 ± 5.00^*1, $2,°3^	0.9 ± 0.4^°3^	8.6 ± 2.2^°3^	0.77:0.02:0.22	0.25 ± 0.05
2) Glucose 45 g/l, 10 g/l YE, 7.5 g/l soya	1.1 ± 0.3 × 10^10^	4.4 ± 0.3^$3,&3^	0.10 ± 0.01^&2^	4.79 ± 0.76^&1^	0.48 ± 0.03^&2,$1^	20.7 ± 2.3^$2,&1^	2.4 ± 0.9^&2^	10.8 ± 0.4^@2,&3^	0.56:0.07:0.37	0.17 ± 0.04
3) Glucose 35 g/l, 10 g/l YE, 7.5 g/l soya	1.2 ± 0.4 × 10^10^	4.3 ± 0.23^*3,§3^	0.14 ± 0.04	4.77 ± 1.70^§1^	0.63 ± 0.11^§2^	20.2 ± 6.6^*1,§1^	0.9 ± 0.4^§3^	5.8 ± 1^@2,§1^	0.56:0.10:0.33	0.23 ± 0.03
3) Fructose 35 g/l, 10 g/l YE, 7.5 g/l soya	5.3 ± 1.3 ×10^9^	2.6 ± 0.2^°3,&3,§3^	0.19 ± 0.02^°1,&2^	1.86 ± 0.23^§1,&1,$2^	0.33 ± 0.06°^2,§2,&2^	4.9 ± 0.9^§1,&1,°3^	4.8 ± 0.3^§3,&2,°3^	1.6 ± 0.3^§1,&3,°3^	0.39:0.42:0.19	n.d.

Data were analysed by one way Anova with post hoc corrections, and results are indicated as apex to the values reported in the table. Comparisons: Gluc 60 vs. Gluc 45, $; Gluc 60 vs. Gluc 35, *; Gluc 60 vs. Fruc 35, °; Gluc 45 vs. Gluc 35, @; Gluc 45 vs. Fruc 35, &; Gluc 35 vs. Fruc 35, §. *p* < 0.05, 1; *p* < 0.01, 2; *p* < 0.001, 3.

### 3.2 Isolation of potentially bioactive fractions

The purification strategy applied in this work was performed on about 2 l of broth recovered from batch fermentations on medium 3 containing glucose. During the ultrafiltration of the supernatant the TMP stayed constant at 0.2 bar and on average the flux decreased from about 14 to 12 l/m^2^ h resulting in a process time of about 60 min. After ultrafiltration, carbon treatment and precipitation, the obtained powder was characterized to quantify the amount of polysaccharides, proteins and water. The same strategy was applied to the sterile medium, as control, to estimate the amount of carbohydrates present in the complex nitrogen sources that could contaminate supernatant samples. Results are reported in Table 2. The solids recovered from the purifications of the fermentation supernatant contained about 40% carbohydrates and 20% proteins. The solids recovered by applying the same purification protocol to the unspent sterile medium accounted for less than 10% of those obtained from *L. brevis* supernatants; this fraction was composed by 40% of carbohydrates, 1.4% proteins and 1.3% water (Table 3).

**TABLE 3:** Quantification of exopolysaccharides, proteins and water in the partially purified supernatant obtained from *L. brevis* SP48 fermentations on medium 3 with glucose as main carbon source. Data recorded from the characterization of powders obtained from two downstream processes of two different batch processes were used to calculate average values.

	Volume (l)	Solids recovered (g)	EPS (g)	Proteins (g)	Water (g)
*L. brevis* supernatant*	2.05 (±0.07)	0.92 (±0.05)	0.39 (±0.08)	0.19 (±0.04)	0.087 (±0.004)
Sterile medium	2	0.07	0.028	0.001	0.001

*The sample was also analysed by HPLC to verify the absence of acids (e.g. Lactic acid).

### 3.3 Characterization of EPS by NMR, GC-MS and HPAE-PAD

**FIGURE 1:**
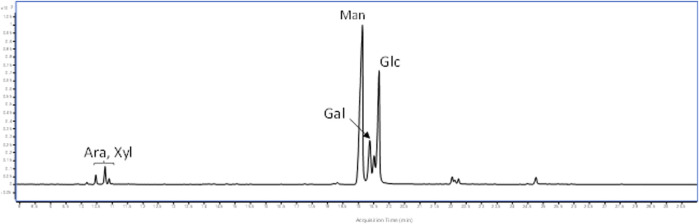
GC-MS chromatogram of acetylated *O*-methyl glycosides from *L. brevis* EPS.

**FIGURE 2:**
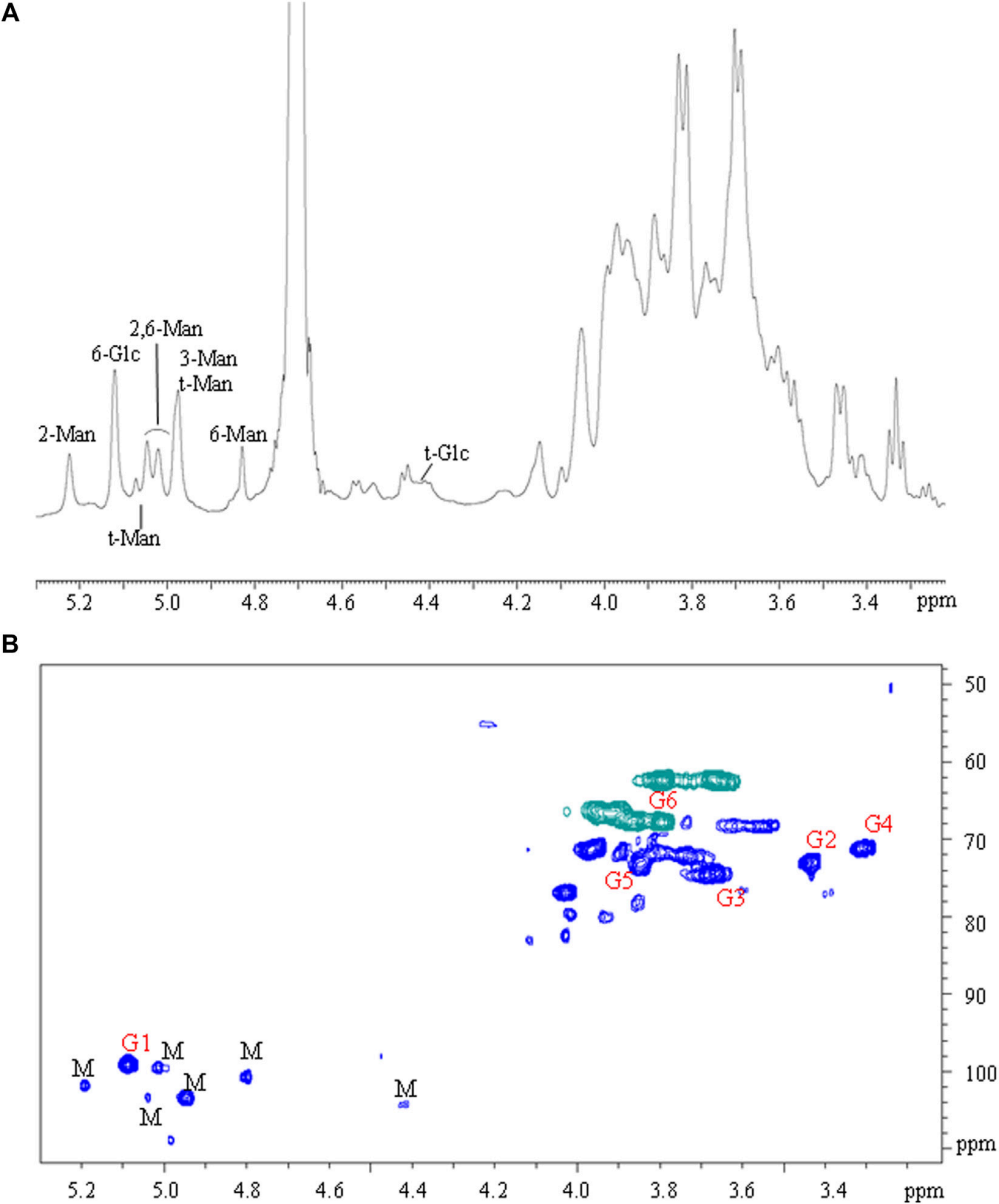
**(A)** ^1^H NMR and **(B)** ^1^H, ^13^C DEPT-HSQC spectra of EPS from *L. brevis.* Letters “M” and “G” refer to mannan and glucan signals, respectively.

The polysaccharides were subjected to methanolysis which resulted in the formation of *O*-methyl glycosides. After the acetylation, the mixture was injected into the GC-MS. The chromatogram indicated the presence of mannose (Man), galactose (Gal), and glucose (Glc) as the main components. Furthermore, traces of xylose (Xyl) and arabinose (Ara) were found (Figure 1). The monosaccharides were identified based on their mass spectra and the retention times on the GC column as described in the Materials and Methods. The methylation analysis of EPS indicated the attachment points of the monosaccharides and revealed the occurrence of terminal glucose, terminal galactose, terminal arabinose, terminal xylose, 6-substituted glucose, 6-substituted mannose, 2,6-disubstituted mannose, and 3,6-disubstituted galactose. In agreement with the different abundance of the monosaccharides, the study of NMR spectra, revealed that the EPS is constituted by a mixture of polysaccharides (Figure 2A). Compatible with the linkage types, in the ^1^H,^13^C DEPT-HSQC spectrum (Figure 2B) signals of anomeric protons attributable to mannose units of an α-mannan were identifiable. The ^13^C chemical shifts were identified by comparing them with those of the unsubstituted residue (Bock and Pedersen 1983). Indeed, the anomeric signals of the residues of the mannan were found at δ 5.19/101.8 (2-Man), 5.04/103.3 (t-Man), 5.01 and 4.99/99.5 (2,6-Man), 4.93/103.4 (3-Man), 4.79/100.7 (6-Man), and 4.37/103.2 (t-Glc) (Casillo et al., 2021). In addition, signals indicating a α-(1–6)-dextran (Mclntyre and Vogel, 1991) were present (Figure 2B and Table 4). Finally, the remaining signals can be attributed to an heteropolysaccharide containing galactose, arabinose, and xylose.

**TABLE 4:** ^1^H and ^13^C NMR chemical shifts of the dextran of the EPS from *L. brevis* SP48.

	Chemical shifts(ppm)					
	H1	H2	H3	H4	H5	H6a,b
	C1	C2	C3	C4	C5	C6
α-(1–6)-dextran	5.09	3.43	3.67	3.30	3.84	3.79/3.86
G	99.1	73.0	74.5	71.0	73.0	67.6

The solid fraction recovered after the downstream process based on UF/DF and ethanol/acetone precipitation, and containing the exopolysaccharide released in the culture medium during cultivation was also hydrolyzed using acidic conditions and analyzed by HPAE-PAD to obtain the main monosaccharides compositions. To better evaluate differences with the polysaccharides eventually present in the medium components, the medium itself was also treated and analyzed using the same protocols. In particular, the three most abundant monosaccharides revealed by the previous GC-MS/NMR experiments were quantified indicating that the broth supernatant derived sample, despite showing the same sugar components of the medium itself, presented a major abundance of galactose and especially glucose in respect to mannose (Cell-free treated supernatant: Galactose_2.6_: Glucose:Mannose; Medium: Galactose_1.8_:Glucose_0.6_:Mannose). These data, although indicating that the polysaccharide fraction also contains some residues of the medium components, ensures a *de novo* synthesis of exopolysaccharides from the strain. To further investigate this, small aliquots of biomass were extracted using previously reported protocols (Tallon et al., 2003), and the polysaccharides derived analyzed as previously described, reported a molar percentage of glucose of 84% and 12% of galactose with residual mannose.

### 3.4 Evaluation of the effects of *L. brevis* SP48 or of derived potential postbiotics against *H. pylori infection in an AGS based in vitro model*

**FIGURE 3:**
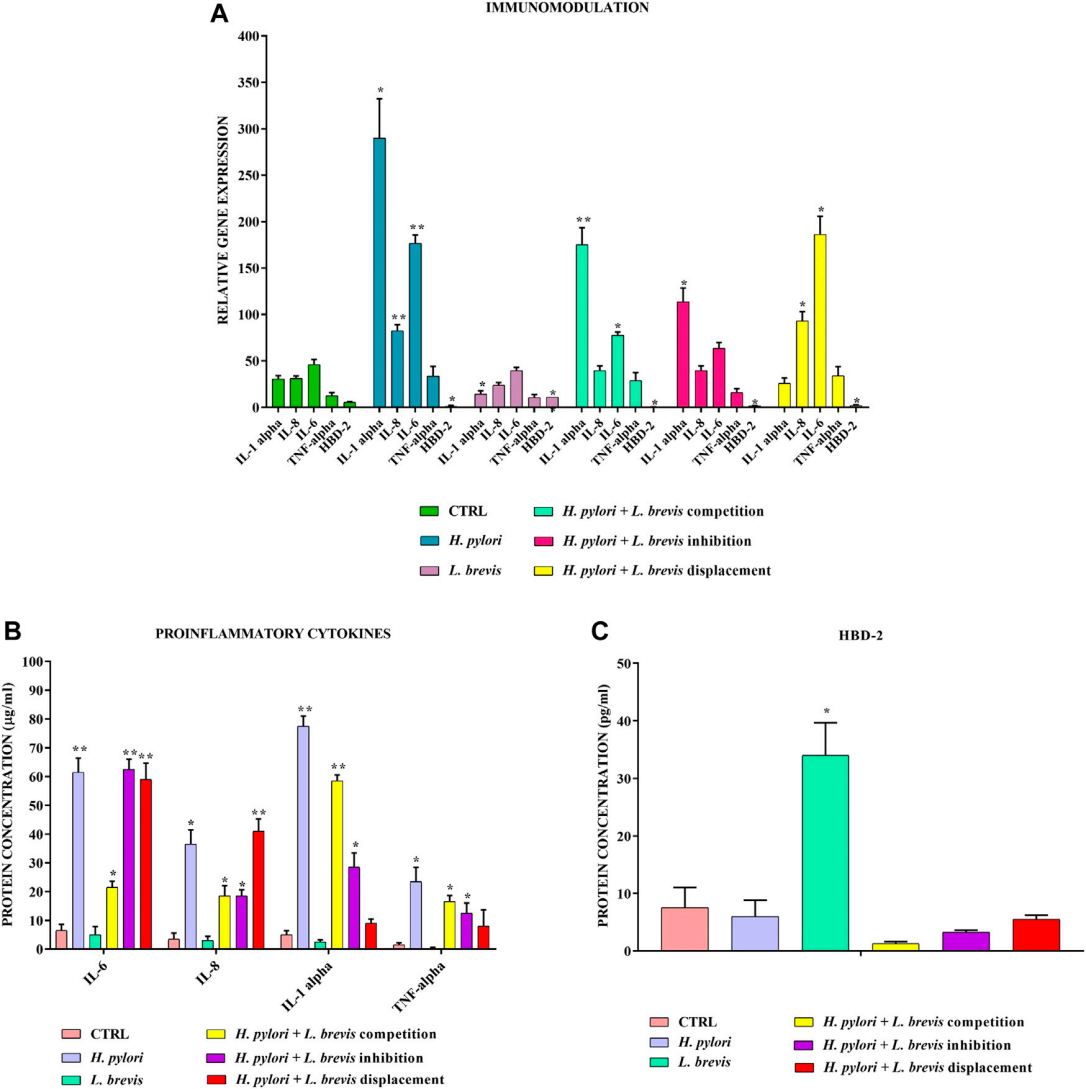
Real-Time PCR **(A)** and ELISA **(B,C)** show the expression levels of pro-inflammatory cytokines and HBD-2 in AGS cells infected with *H. pylori* and/or *L. brevis* SP48. Data are expressed as relative mRNAs expression **(A)** and protein concentration **(B,C)** in each group and are representative of three different PCR and ELISA experiments ±SD. RNA was extracted from 3 different AGS cell samples treated with the same batch of *H. pylori* and *L. brevis*. Significant differences are indicated by *p < 0.05, **p < 0.01, and ***p < 0.001.

The infection to the AGS cells with *H. pylori* was carried out in three different ways to better understand in which condition *L. brevis* best expresses its anti-inflammatory properties. The data obtained show that *L*. *brevis* added before, simultaneously or after *H. pylori*, significantly reduces the inflammatory state induced in AGS cells. In fact, the genes encoding proinflammatory cytokines IL-1α, IL-6, TNF-α, and IL-8 are strongly downregulated compared to infection with *H. pylori* alone (Figure 3A). The expression of related proteins was evaluated by ELISA assay and confirmed these data (Figure 3B). Only anti-inflammatory cytokine TGF-β is unmodulated. Therefore, *L. brevis*, which alone can reduce the basal expression of proinflammatory molecules, is also able to increase anti-microbial defenses by inducing the expression of HBD-2 (Figure 3C), thereby improving the conditions of the gastric mucosa damaged by *H. pylori*.

**FIGURE 4:**
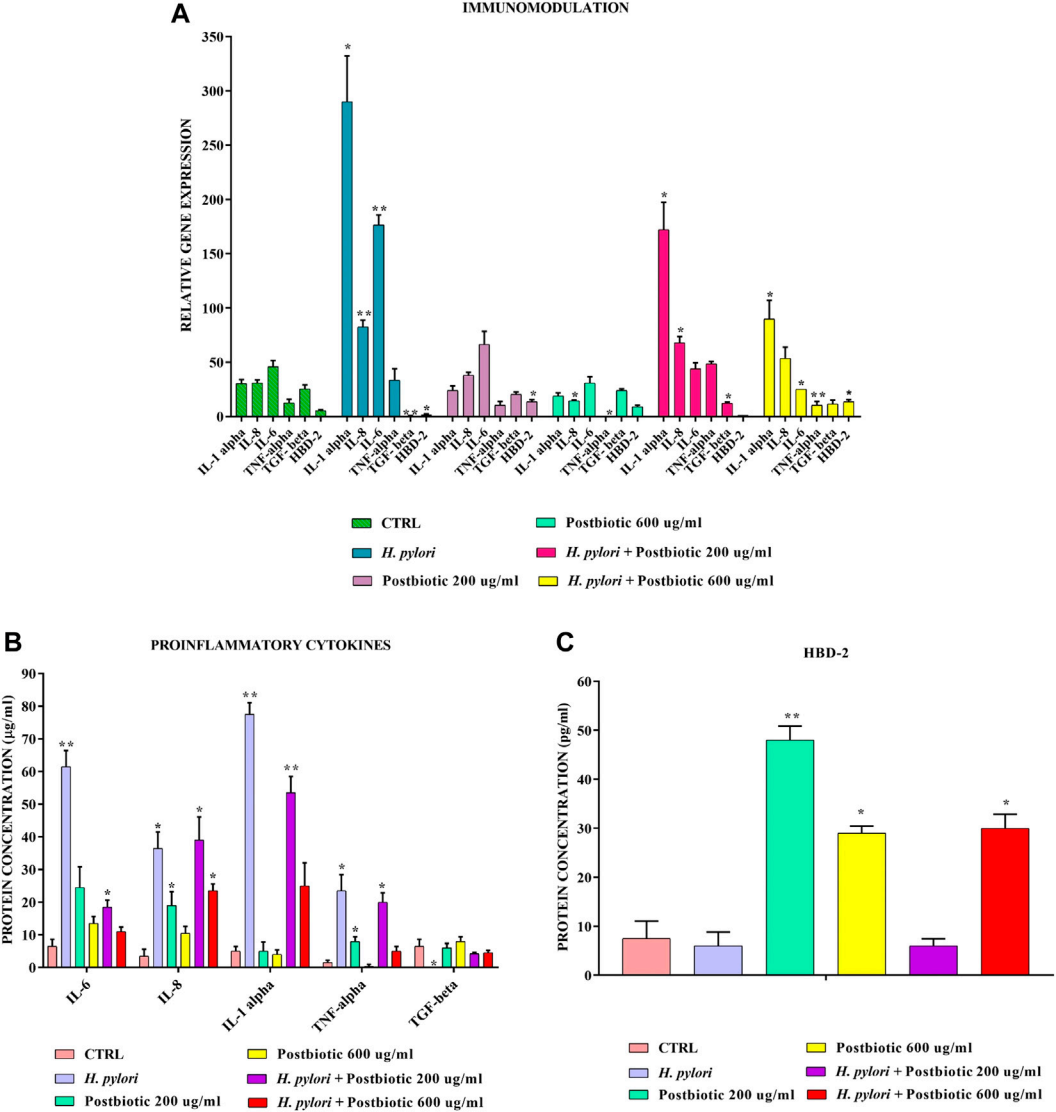
Real-Time PCR **(A)** and ELISA **(B,C)** show the expression levels of proinflammatory cytokines and HBD-2 in AGS cells infected with *H. pylori* and/or potential postbiotics of *L. brevis* SP48. Data are expressed as relative mRNAs expression **(A)** and protein concentration **(B,C)** in each group and are representative of three different PCR and ELISA experiments ±SD. RNA was extracted from 3 different AGS cell samples treated with the same batch of *H. pylori* and postbiotics. Significant differences are indicated by *p < 0.05, **p < 0.01, and ***p < 0.001.

The same model (Figure 4A–C) showed that postbiotics of *L. brevis* found in the supernatant after 16 h of growth can also significantly downregulate at both tested concentrations the expression of pro-inflammatory cytokines IL-1α, IL-6, TNF-α, and IL-8 and upregulate the expression of anti inflammatory cytokine TGF-β after infection with *H. pylori*. In addition about 200 μg/ml of the fraction are already sufficient, to induce the expression of HBD-2.

### 3.5 Assessment of inflammation biomarkers on human enterocytes challenged with LPS and treated with *L. brevis* SP48 or secreted potential postbiotics: TLR-4 activation and IL-6 mRNA expression

**FIGURE 5:**
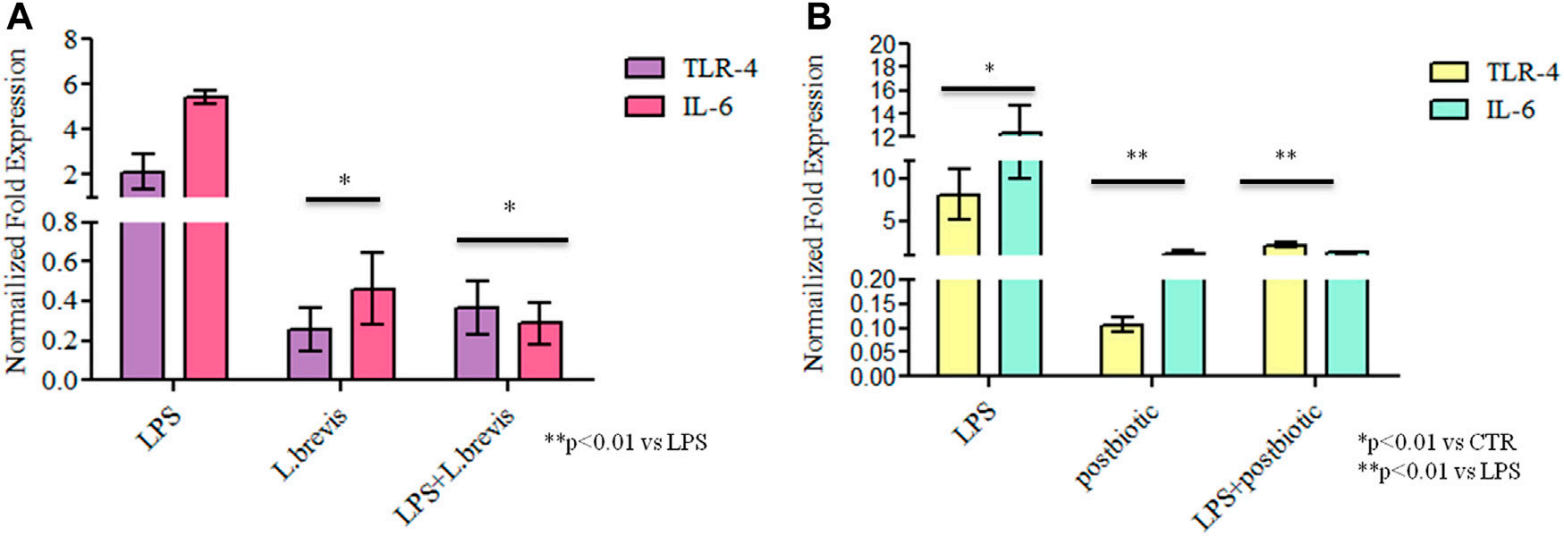
Gene expression analyses: results are expressed as fold change of **(A)** LPS, *L. brevis* alone and LPS + *L. brevis* and **(B)** LPS, postbiotic, and LPS + postbiotic treated cells in respect to untreated cells (CTR) for TLR-4 and IL-6 in human enterocytes. RNA was extracted from two different wells treated with the same batch of samples. Data are shown as average ±SD. Student t-test was used for the evaluation of statistical differences and significance was indicated as *p < 0.01 vs CTR, **p < 0.01 vs LPS.

In order to evaluate the anti-inflammatory efficacy of *L. brevis* alone and in combination with LPS, the transcriptional level of TLR-4 and IL-6 gene expression was analyzed *in vitro* on a cell based model (Figure 5A). Both TLR-4 and IL-6 were upregulated, as expected, in the presence of LPS from *S. minnesota*. TLR-4 expression was significantly reduced, respect to the CTR, when enterocytes were treated with the sole *L. brevis*, when the probiotic strain was added to LPS treated cells TLR-4 was strongly reduced (about 9 and 5 fold less). Also, the expression level of IL-6 with the sole *L. brevis* and after the insult with LPS, determined a significant reduction (12 and 20 fold less) *versus* LPS treatment (*p* < 0.01). In fact, both biomarkers showed a similar, or even diminished expression when compared to untreated cells. As shown in Figure 5, TLR-4 expression was significantly reduced even in respect to the CTR, when enterocytes were treated with the sole postbiotics, and the reduction was very high when compared to LPS treatment (about 75 fold less). When postbiotics were added in combination to LPS, they proved to reduce the detrimental effects of the LPS that caused upregulation of the inflammation biomarkers tested. In fact, with LPS treatment, IL-6 increased its expression of about 12 fold vs. CTR. The expression level of IL-6 with the sole postbiotics from *L. brevis* was similar to CTR; the combination of postbiotics and LPS, caused a marked reduction of the expressions of the 2 markers in respect to LPS insulted cells corresponding to a 1.8 and a 3 fold reduction for IL-6 and TLR-4, respectively, with respect to the LPS treatment (*p* < 0.01).

### 3.6 NF-κB mediated response, in an enterocytes model, in the presence of *L. brevis* SP48 or of secreted potential postbiotics alone or after LPS treatment

**FIGURE 6:**
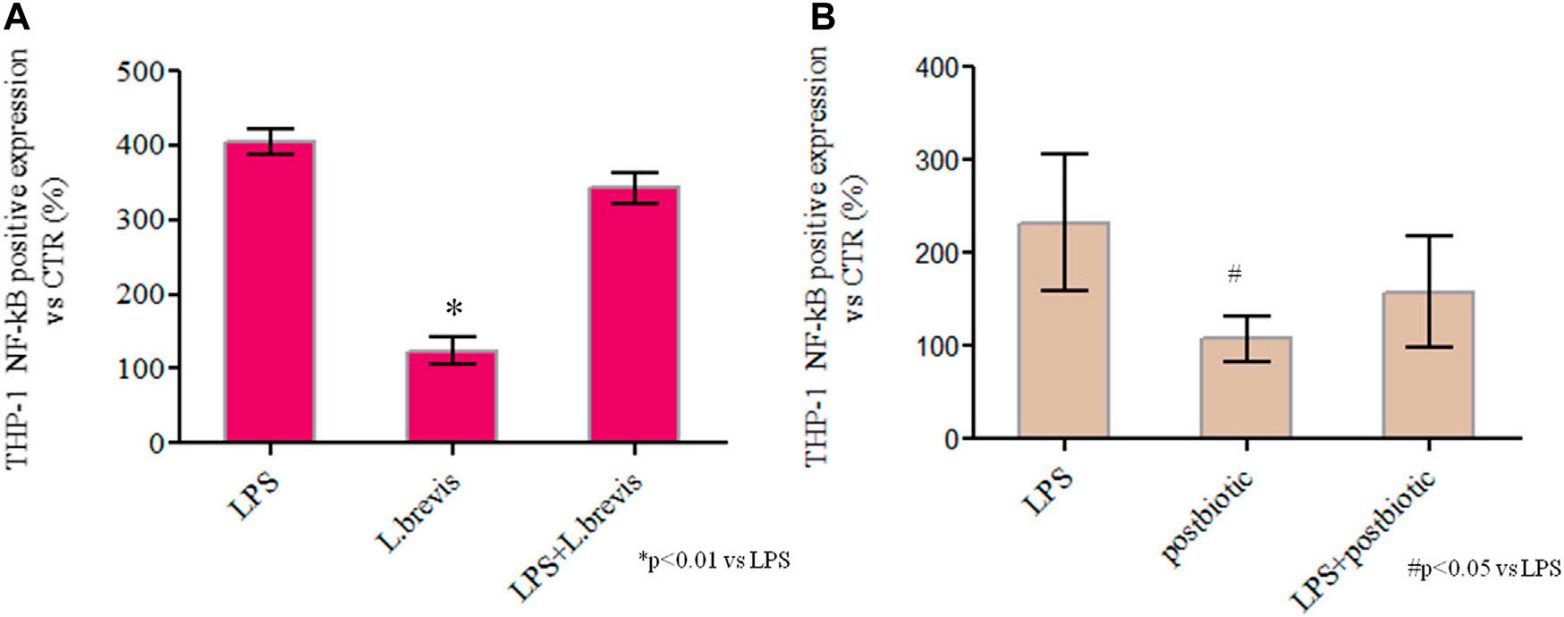
Spectrophotometric quantification of NF-κB by QUANTI-Blue™ assay in untreated cells (CTR) or cells exposed to commercial LPS from *Salmonella*, *L. brevis*, and potential postbiotics secreted from *L. brevis* and partially purified as described in the text. **(A)** Cells treated with LPS, LPS + *L. brevis,* and *L. brevis* alone **(B)** Cells treated with LPS, LPS + postbiotics and postbiotics alone **(B)**. The assay was performed in duplicate, cells were treated with the same batch of postbiotics. The results are shown as average ± SD and normalized with respect to CTR (%). Student t-test was used for the evaluation of statistical differences and indicated as *p < 0.01 and #*p* < 0.05 vs. LPS.

**FIGURE 7:**
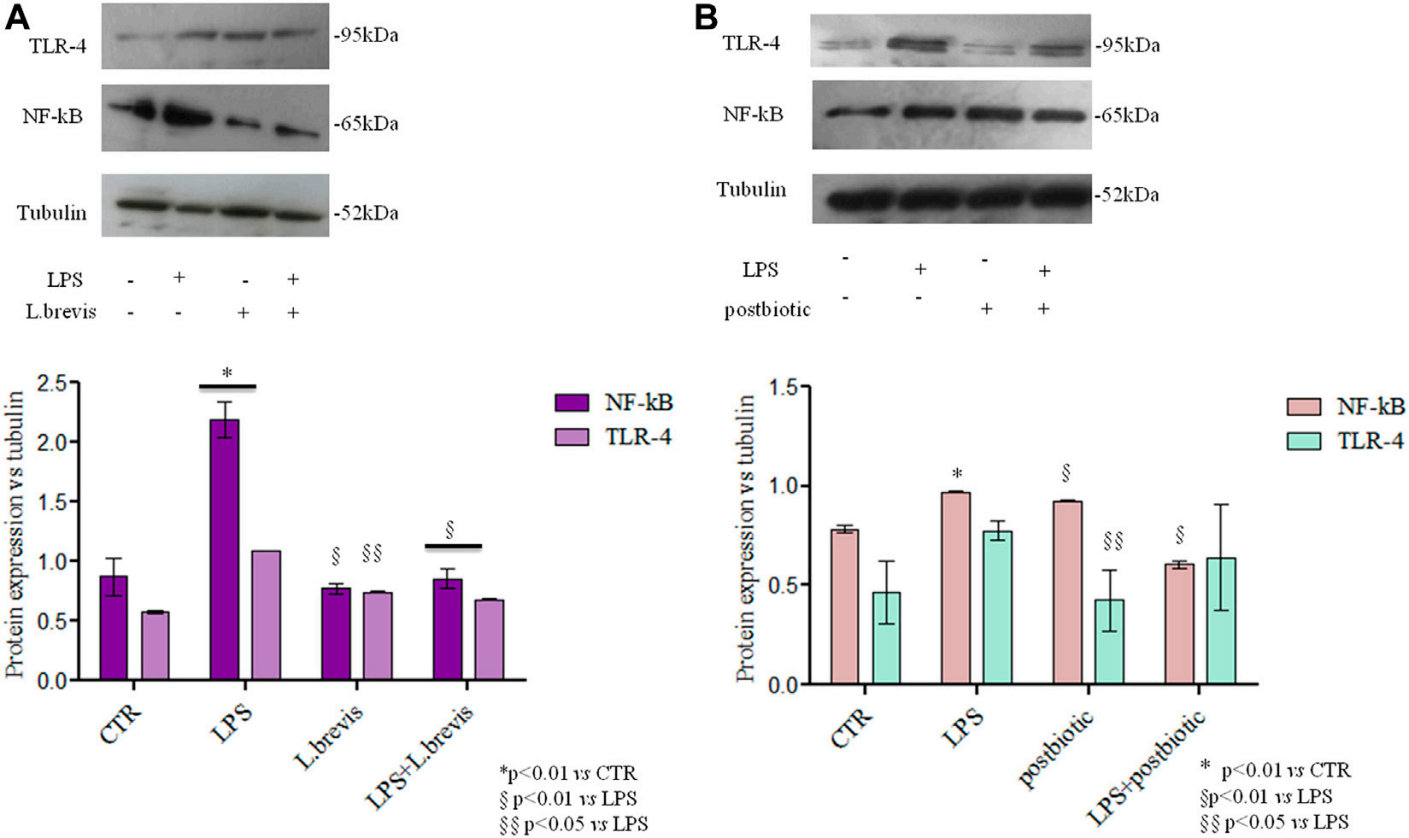
TLR-4 and NF-kB protein expression analyzed by western blotting in differentiated Caco-2 cells treated with **(A)** Cells insulted with LPS were treated with *L. brevis* **(B)** Cells insulted with LPS were treated with postbiotics secreted by *L. brevis* (and partially purified as described in the text). The intensity of the bands was normalized in respect to tubulin in the densitometry reported as average and SD. Student t-test was used for the evaluation of statistical differences and indicated as *p < 0.01 vs. CTR, §*p* < 0.01 vs. LPS and §§*p* < 0.05 vs. LPS.

A recently proposed colorimetric assay based on the detection of NF-kB activation in monocytes was also used in the framework of this study. The results further highlighted the activation of the inflammation pathway (through THP1-Blue™ cells) when enterocytes were challenged with LPS. In fact, the co-culture of differentiated Caco-2/THP1-Blue™ NF-κB reporter cells allowed to evaluate the inflammatory modulation *via* the NF-κB pathway comparing the response of enterocytes to the addition of *L. brevis* live cells and postbiotics alone, or upon their addition after the challenging of cells with LPS to induce inflammation. As reported in Figure 6 the LPS treatment upregulates NF-kB with respect to the CTR. The treatment with *L. brevis* and postbiotic fraction (Figure 6A) was similar to the control. The addition of either *L. brevis* or postbiotics to LPS treated cells on average slightly reduced the NF-kB level (Figure 6A). These data indicated an anti-inflammatory response of both *L. brevis* SP48 cells and secreted compounds (potential postbiotics). However, since results were not statistically significant, to identify and confirm the underlying signaling pathways, TLR-4 and NF-κB activation were evaluated by western blotting experiments in protein extracts derived from treated enterocytes (Figure 7). TLR-4 expression slightly increased with respect to the control, in LPS treated cells. However, addition of *L. brevis* to LPS challenged cells significantly decreased TLR-4 expression to levels similar to those of untreated cell. LPS markedly increased NF-kB expression of about 2 fold with respect to untreated cells. Enterocytes treated with LPS showed, upon addition of *L. brevis*, a 2.8 fold lower NF-kB level compared to the LPS treated cells (Figure 7A).

The same experiment was performed to evaluate the potential postbiotic fraction secreted by *L. brevis* SP48. Unexpectedly, addition of the sole postbiotics resulted in slightly higher NF-kB expression levels. However, addition of the postbiotics to LPS challenged cells significantly reduced the expression of NF-kB. TLR-4 expression, on the contrary, was not significantly reduced in LPS challegend cells treated with the postbiotic fraction due to high data variability (Figure 7B).

## 4 Discussion

Probiotic strains have been demonstrated to provide several health benefits that are due to their specific interaction with the host and/or with eventual pathogens, or to the production of a variety of molecules with critical functions/roles. Several *L. brevis* strains have been recognized to modulate the immune system and affect human health (Fang et al., 2018; Prado Acosta et al., 2021). Therefore, not only the production of live cells but also the identification of potentially bioactive molecules, such as exopolysaccharides, produced from *L. brevis* strains during growth, is of great interest. To this end, this work focused on the identification of a fermentation medium that could support production of biomass and bioactive metabolites from *L. brevis*, and at the same time allow the application of a simple downstream process to obtain a potentially bioactive fraction and characterize it from a biological (and structural) point of view.

The use of De Man, Rogosa and Sharpe (MRS) medium is still quite common for the growth of *Lactobacillus* also in bioreactor experiments (Yue et al., 2013; Song et al., 2021). In previous research studies aeration combined with an exponential feeding profile on MRS medium resulted in the production of about 9 x10^9^ CFU/ml of *L. brevis* CD2 in 42 h of growth (Alfano et al., 2020).

On the other hand, renewable waste materials were conveniently used as substrates for the development of processes targeting the recovery of products secreted by different *L. brevis* strains (e.g. antimicrobial molecules, mannitol, GABA etc) (Li et al., 2010; Yue et al., 2013; Xie et al., 2019; Gubelt et al., 2020).

Obtaining high cell densities during fermentation and further processing, is crucial to ensure the delivery of an adequate number of viable cells in the target area, for potential industrial purposes. Only few reports describe the optimization of *L. brevis* fermentations, and titers of viable cells are often not reported (Haixing et al., 2010; Mårtensson et al., 2003). In this study a semi defined medium deprived of components of animal origin allowed to obtain the highest concentration of viable cells (10^10^) described up to date and about 200 mg/L of exopolysaccharides in a simple 16 h batch experiment. A cheap medium with a high concentration of glucose, yeast extract and soy peptone was investigated to achieve suitable biomass yields and productivities, thereby shortening process time. However, the presence of polysaccharide contaminants in complex nitrogen sources complicates the purification of potentially bioactive compounds, such as exopolysaccharides produced by lactobacilli, and the characterization of their structure (Ferrari et al., 2022). To define a simpler medium and an industrially feasible purification strategy, the effect of lower concentrations of complex nitrogen sources (and glucose) was evaluated in the same experimental set-up demonstrating, unexpectedly, that the reduction did not affect the titer of viable cells and of EPS produced. Although higher glucose and complex nitrogen concentrations corresponded to improved dry biomass production, the Y_x/s_ and the concentrations of viable cells did not change significantly. This might partly be due to the toxicity of lactic acid that doubled on the richer medium reaching a titer of about 40 g/l. *L. brevis* was previously demonstrated to possess the complete PTS system and the enzymes that carry out the first steps of glycolysis; these enzymes are fructose inducible and oxygen repressible (Saier et al., 1996; Guo et al., 2017). Growth on glucose in anaerobic conditions, confirmed previous results indicating the production of lactate and ethanol, mainly, with trace amounts of acetate (Guo et al., 2017). Higher glucose concentrations favored ethanol production to the detriment of LA (medium 2 vs. medium 3), whereas the concurrent increase of glucose and complex nitrogen sources in the medium (medium 1, Table 2) re-established a higher LA:Ethanol molar ratio. Growth on fructose in the same conditions yielded equimolar amounts of lactic acid and acetic acid, as expected, probably due to oxidation of fructose to carbon dioxide and ribulose-5P *via* the heterolactic fermentation, and the formation of acetate from acetyl phosphate catalyzed by acetokinase that produces additional ATP (Guo et al., 2017). Differently from other strains (Neveling et al., 2012; Alfano et al., 2020), compared to glucose, growth of *L. brevis* SP 48 on fructose was less efficient resulting in lower biomass and lactic acid titers, and interestingly no exopolysaccharide was detected in the fermentation broth in the assayed conditions, probably indicating an overall lower concentration of polymer produced. The use of D-fructose as carbon source for *L. brevis* TMW 1.2112, although enabling fastest and strongest growth compared to other sugars, resulted in the lowest slime formation (Fraunhofer et al., 2018). This strain is known to produce a β-glucan, and the conversion of D-fructose to UDP-glucose, the polysaccharide precursor, requires three metabolic steps that consume 2 ATP molecules, in contrast to consumption of 1 ATP and two metabolic steps for the conversion of Glc-1-P from glucose. The authors suggested that instead of producing high quantities of β-glucan-cell networks, D-fructose was probably rather directed to the glycolysis for generating energy (Fraunhofer et al., 2018). Therefore, for *L. brevis* TMW 1.2112 fructose does not induce/is not preferred for β-glucan biosynthesis; it seems that similarly here fructose does not promote EPS production. Fraunhofer and colleagues (2018) demonstrated that the type of sugar (maltose, glucose, fructose, galactose, ribose, arabinose, xylose, melibiose) used as carbon source strongly impacts the concentration of polysaccharide produced, whereas it has no effect on the composition of the EPS. Also for other LAB species a strong correlation between the concentration of EPS produced and the carbon source was previously described (Padmanabhan et al., 2018).

EPS titres here obtained on the simpler medium (0.25 g/ll) were in the range with those observed in other studies that report the production of about 0.16 g/l and 0.13 g/l from *L. brevis* MSR 104 (Riaz Rajoka et al., 2020) and *L. brevis* CD2 (Alfano et al., 2020), in batch process on MRS medium.

The compounds secreted by probiotics (e.g. polysaccharides, proteins, fatty acids, peptides, and organic acids) are biologically active molecules that often optimize physiological and immune functions of the host and provide numerous benefits. Cell-free supernatants of lactobacilli have frequently been shown to possess antimicrobial properties due to the presence of organic acids and bacteriocins (Yang et al., 2021; Moon et al., 2022). The supernatants obtained from the growth of *L. brevis* SP 48 were here investigated to study the effects of a polysaccharide- and protein-enriched secreted fraction, deprived of organic acids and bacteriocins removed by ultrafiltration.

The NMR and GC/MS analyses highlighted the presence of different polysaccharides, mannan prevalently coming from the medium (possibly from yeast extracts), galactan and glucan, in this last case an α-glycosidic linkage was found. Further structural features were difficult to assess due to the presence of multiple species and of neutral polymers (no amines and no uronic acid residues were found). However, from the monomers quantification it was possible to highlight that the biosynthesis of the strain studied regarded principally glucans and possibly a lower amount of galactose based polymers (or glucose/gal) ones. Overall, these data agree with the scientific literature dealing with EPS from diverse *L. brevis* strains.

Since yeast extract was present in the growth medium, the sterile initial fermentation medium underwent the same purification protocol to demonstrate that only a portion of the bioactive fraction may derive from medium components. The two solid powders obtained from the downstream protocol, the one recovered from the sterilized medium and the one here considered as potential postbiotic, were also hydrolysed, and analysed by HPAE-PAD to quantify the three main sugar components (glucose, mannose and galactose) found by NMR/GC-MS analysis. Data indicated a prevalence of galactose in both samples and of glucose over mannose in the cell-free supernatant. This suggests the presence of one or more polysaccharides in the samples, besides those deriving from yeast extract, with glucose and galactose as main components. Extraction of the cell-bound EPS from the biomass of *L. brevis* SP48 indicated the presence of a glucose rich polysaccharide confirming *de novo* biosynthesis of EPS from the strain.

Cell-free supernatants of *L. brevis* (strain MK757902) have previously been shown to exhibit anti-cancer properties (Park et al., 2015). In particular, the partially purified proteins from the spent culture supernatant of *L. brevis* MK05 induced apoptosis of MCF-7 cancerous cells (Pourbaferani et al., 2021). Sasaki and collaborators demonstrated that heat-killed *L. brevis* KB290 and crude EPS significantly enhanced cytotoxic activity of mouse splenocytes, and, that the EPS demonstrated to have a critical role in enhancing cell-mediated cytotoxic activity in mouse spleen (Sasaki et al., 2015). Since *L. brevis* CD2 was previously shown to reduce the intragastric load of *H. pylori* (Linsalata et al., 2004) the effect of *L. brevis* SP48 viable cells and of the potentially bioactive compounds on the inflammatory state of the intestinal epithelium during *H. pylori* infection was here evaluated. Competition, inhibition, and displacement assays on AGS cells treated with *L. brevis*/potential postbiotics alone or following *H. pylori* infection were performed. The experiment was carried out in 3 different ways to better understand the conditions in which *L. brevis* best exerts its inhibitory activity against *H. pylori*. First, by adding the *L. brevis* before *H. pylori*, its production of bacteriocins and other antimicrobial factors could inhibit the growth of *Helicobacter* by decreasing its concentration and therefore its power to adhere to the cells; the simultaneous addition of the two species, on the other hand, could lead to a competition both for the intake of nutritional factors (which would necessarily cause one species to prevail over the other) and for the binding sites to the receptors present on the surface of the epithelial cells. Finally, the addition of *L. brevis* after infection with *H. pylori* could cause a displacement of the latter from the binding sites, again owing to the production of antimicrobial factors capable of causing its killing. On the other hand, it is know that *L. brevis* and other *lactobacillus* strains are able to modulate immune responses of the host by regulating the expression of proinflammatory cytokines, soluble mediators of natural immunity (Kanmani and Kim, 2020), and of Human β-defensin-2 (HBD-2), inducible antimicrobial peptide active against Gram-positive and Gram-negative bacteria, fungi, and the envelope of some viruses, and involved in the innate immune response (Kanmani and Kim, 2020; Donnarumma et al., 2016). Our results showed that *L. brevis* alone and the secreted compounds at both concentrations increased the production of HBD-2 by gastric epithelial cells indicating the stimulation/activation of antimicrobial defenses and of innate immunity. Concurrently both live cells and postbiotics by themselves strongly downregulated the expression of proinflammatory cytokines caused by *H. pylori* infection, demonstrating an anti-inflammatory effect. These data demonstrated that the high molecular weight potentially postbiotic fraction (mainly containing exopolysaccharide and proteins) secreted by *L. brevis* is by itself sufficient to contrast *H. pylori,* thereby overcoming issues related to the preservation of probiotic viability.

The fact that substantial differences in the results obtained in the 3 different tests are not appreciated, could mean that at the basis of the inhibition of *H. pylori* mediated by *L. brevis*, different mechanisms are involved simultaneously, including those previously hypothesized, but it is also interesting to underline how this behavior is species-specific, in fact, as observed in our previous work (D’Ambrosio et al., 2022), using viable cells of *L. fermentum* the inhibition of *H. pylori* proved efficient only in the displacement assay.

*L. brevis* SP48 and its secreted high molecular weight fraction was also tested *in vitro* in a gut model. Recent literature reports that extracellular polysaccharides modulate intestinal immune responses by regulating Toll-like receptors (e.g TLR-2 and 4) signaling pathways, which are responsible for the induction of cytokines and chemokines in response (Wachi et al., 2014).

*In vitro* cell models play an important role in understanding cellular mechanisms related to the function of specific molecules. Lactic acid bacteria showed anti-inflammatory properties activating a non-specific response of the immune system (El-Deeb et al., 2018). They are also known to play a significant role in activating NF-kB, which regulates the production of pro-inflammatory cytokines in LPS-stimulated cells (Lee et al., 2008; El-Deeb et al., 2018).

In the present study cells challenged with LPS from *S. minnesota* showed upregulation of all the inflammatory biomarkers tested. The treatment with *L. brevis* SP48 reduced the mRNA expression of TLR-4 and IL-6 like pro-inflammatory targets, as well as the protein expressions of TLR-4 and NF-kB. These data suggested that *L. brevis* SP48 exerts a direct action on the human enterocytes stimulating the immune response and inactivating the NF-κB inflammatory pathway. Interestingly, also the bioactive compounds isolated from *L. brevis* supernatants acted as modulators of the inflammatory pathway by reducing the expression of inflammatory mediators in the presence of LPS. In fact, upon LPS addition the NF-κB pathway was activated through TLR-4 binding and prompted cytokines production as already described in the recent literature (Nyati et al., 2017; Di Guida et al., 2021). The potential postbiotics isolated in this work reduced pro-inflammatory cytokines (e.g. IL-6), by decreasing expression/activation of NF-kB and TLR-4, thereby indicating that they can modulate the enterocytes’immune response (Di Guida et al., 2021). The THP-1 blue NF-κB reporter assay indicated an inhibitory effect of *L. brevis* SP48 secreted compounds on inflammation in response to LPS binding. Western blotting experiments confirmed the downregulation of NF-kB upon treatment of LPS challenged cells with the postbiotic fraction. Also other exopolysaccharides reduced the secretion of cytokines and downregulated the NF-κB pathway (El Deeb et al., 2018; Chen et al., 2019; Liu et al., 2017) demonstrating appealing anti-inflammatory properties, for the potential treatment of the intestinal bowel disease (e.g. IBD) without the need of live cells.

## Conclusion

The optimization of fermentation processes and the development of economically viable probiotic production processes is necessary to reduce costs of commercial products. On the other hand, several probiotic strains secrete a plethora of biologically active molecules that by themselves exert beneficial effects and can be useful in the medical and food fields. Results presented in this study demonstrate the production of high titers of viable *L. brevis* SP48 cells on a cheap medium deprived of animal derived raw materials, and at the same time the obtainment of a biologically active cell-free fraction. Live cells and secreted compounds reduced inflammation of gastric epithelial cells following *H. pylori* infections. The high molecular weight compounds present in the fermentation supernatant also modulated the expression of inflammatory mediators in an *in vitro* gut model. These effects seem to be due, at least in part, to EPS and/or secreted proteins other than low molecular weight compounds (e.g. organic acids) that were removed during the ultrafiltration and precipitation processes. These preliminary results indicate that further investigations on *L. brevis* SP48 and on its secreted high molecular weight fraction (potential postbiotic) as potential tools to counteract stomach pathogens and inflammation also in intestinal diseases, are of great interest.

## Data Availability

The original contributions presented in the study are included in the article/Supplementary Material, further inquiries can be directed to the corresponding authors.
